# T Cell Immunosenescence, Hypertension, and Arterial Stiffness

**DOI:** 10.4178/epih/e2014005

**Published:** 2014-05-23

**Authors:** Hee Tae Yu, Eui-Cheol Shin

**Affiliations:** Laboratory of Immunology and Infectious Diseases, Graduate School of Medical Science and Engineering, KAIST, Daejeon, Korea

Immunosenescence is a concept used to describe the age-associated deterioration of immune function, characterized by functional and phenotypic changes on both cellular and systemic levels [1,2]. Immunosenescence in elderly people is associated with an increased susceptibility to infectious diseases, impaired response to vaccinations, and increased incidence of cancer and autoimmunity [3-5]. During immune aging, senescent T cells accumulate in the peripheral T cell pool [2,6,7]. By repetitive antigenic stimulation of T cells, their telomeres are shortened [8] and the expression of CD28, a co-stimulatory molecule, is lost [9]. In humans, cytomegalovirus (CMV) is known to be one of the most important antigens that cause repetitive T cell stimulation [10,11]. As a result of such repetitive stimulation, CD28^null^ T cells accumulate during the immune aging process [12]. Similarly, the expression of CD57 antigen on T cells is currently considered to be another surrogate marker of the replicative senescence of T cells [13]. CD57 is a terminally sulfated glycan carbohydrate epitope, and CD57^+^T cells showed an inability to proliferate after antigenic stimulation *in vitro* and a high susceptibility to activation-induced apoptosis [14,15] (Figure 1). Compared to CD28^+^CD57^-^T cells, CD57^+^ T cells produce more proinflammatory cytokines and exert greater cytotoxicity [16]. These senescent CD57^+^ T cells are known to be associated with a number of inflammatory diseases in humans [17-19].

Recent studies have reported the association between senescent T cells and various cardiovascular diseases. Accelerated telomere shortening in T cells and peripheral blood mononuclear cells (PBMCs) was seen in patients with atherosclerosis [20] and myocardial infarction [21]. Higher frequencies of CD8^+^CD28^null^ CD57^+^ T cells were associated with an increased prevalence of carotid artery lesions in a study of human immunodeficiency virus-infected patients [22]. Liuzzo et al. [23] reported that the number of CD4^+^CD28^null^T cells was significantly greater in the peripheral blood of patients with unstable angina (UA) compared to the number in those with chronic stable angina. The frequency of CD4^+^CD28^null^T cells correlated with the number of interferon (IFN)-γ-secreting T cells, suggesting that CD28^null^ T cells could be the main source of IFN-γ production in patients with UA. Furthermore, it was shown that a higher frequency of CD4^+^CD28^null^T cells was associated with the recurrence of acute coronary events in UA patients [24]. Patients who experienced recurrent acute coronary events had a higher frequency of CD4^+^CD28^null^ T cells than those with a single episode during 4 years of follow-up. Another study found that the number of CD4^+^CD28^null^ T cells is expanded in patients with diabetes mellitus, and this expansion is associated with poor glycemic control [25]. The presence of these senescent T cells also correlates with the occurrence of a first cardiovascular event and with worse outcomes after an acute coronary syndrome.

Senescent T cells have been studied not only in conjunction with atherosclerotic disease but also with hypertension [26]. Patients with hypertension had an increased frequency of circulating CD8^+^ cytotoxic T cells bearing surface markers of replicative senescence, which is characterized by the loss of CD28 and the acquisition of CD57. These individuals also had higher circulating levels of the C-X-C chemokine (CXC) receptor type 3 chemokines, such as CXCL9, CXCL10, and CXCL11. The frequency of CD8^+^ T cells producing perforin, granzyme B, IFN-γ, or tumor necrosis factor (TNF)-α was increased in the peripheral blood of hypertensive patients than in that of healthy controls. However, there was no difference in the frequency of regulatory T cells and IL-17-producing CD8^+^ T cells between the two groups. Immunohistochemical staining of kidney sections from patients with hypertensive nephrosclerosis revealed an increase in CD4^+^ and CD8^+^ T cells, along with greater expression of CXCL11, in the proximal and distal tubules compared with that in normotensive controls.

Arterial stiffening is one of the major mechanisms for the pathogenesis of hypertension [27], and arterial stiffness is increased in the presence of established cardiovascular risk factors including aging [28]. The degree of arterial stiffness is known to be associated with various markers of inflammation [29,30], suggesting a certain role for immunological factors in increasing arterial stiffness. In this regard, we recently studied whether T cell immunosenescence is associated with arterial stiffness evaluated by pulse wave velocity (PWV) in Koreans. We found that the frequency of CD57^+^ cells in the CD8^+^ T cell population was independently correlated with the PWV, even after adjusting for age, sex, systolic blood pressure, diabetes mellitus history, smoking history, body mass index, creatinine, high-density lipoprotein cholesterol, and high sensitivity C-reactive protein (data not published). We also studied CMV-specific T cell responses since CMV is a major driving antigen for the replicative senescence of T cells [10,11]. The frequency of CMV-specific IFN-γ or TNF-α secretion or the cytotoxic function of CD8^+^ T cells was positively correlated with PWV in multivariate analysis (data not published). Recently, the significance of CMV infection in increased arterial stiffness was also reported by Wall et al. [31]. They demonstrated that arterial stiffness was higher in the CMV-seropositive group than in the CMV-seronegative group among patients with chronic kidney disease.

In summary, T cell senescence can be evaluated by the frequency of CD28^null^T cells or CD57^+^ T cells in PBMCs. CMV is a major factor accelerating T cell senescence. T cell senescence is related to cardiovascular diseases such as atherosclerosis, acute myocardial infarction, and hypertension. It is also associated with increased arterial stiffness. The roles of T cell senescence and CMV infection in cardiovascular disease need to be validated in large cohort studies, and the mechanism by which senescent T cells contribute to the pathogenesis of cardiovascular disease requires further investigation.

## Figures and Tables

**Figure 1.**
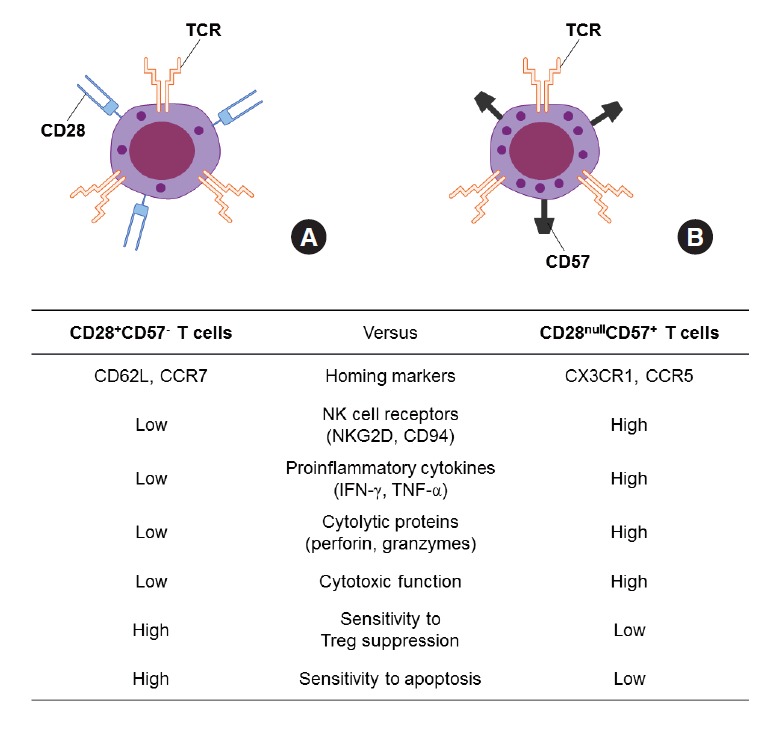
Comparison of CD28^+^CD57^-^ T cells (A) and CD28^null^CD57^+^ senescent T cells (B). INF, interferon; TNF, tumor necrosis factor.
